# A Fiber- and Polyphenol-Enriched Diet Enhances Humoral Immunity, Reshapes Cecal Microbiota, and Improves Short-Chain Fatty Acid Production in Female Wistar Rats

**DOI:** 10.3390/nu18132088

**Published:** 2026-06-26

**Authors:** Sergi Casanova-Crespo, Daniela Ceballos-Sánchez, Anna Vallverdú-Queralt, Maria José Rodríguez-Lagunas, Malen Massot-Cladera, Margarida Castell, Francisco José Pérez-Cano

**Affiliations:** 1 Physiology Section, Department of Biochemistry and Physiology, Faculty of Pharmacy and Food Science, University of Barcelona (UB), 08028 Barcelona, Spain; sergi.casanova@ub.edu (S.C.-C.); daniceballoss@ub.edu (D.C.-S.); mjrodriguez@ub.edu (M.J.R.-L.); malen.massot@ub.edu (M.M.-C.); franciscoperez@ub.edu (F.J.P.-C.); 2 Nutrition and Food Safety Research Institute (INSA), University of Barcelona (UB), 08921 Santa Coloma de Gramenet, Spain; avallverdu@ub.edu; 3 Polyphenol Research Group, Department of Nutrition, Food Science and Gastronomy, Faculty of Pharmacy and Food Science, University of Barcelona (UB), 08028 Barcelona, Spain; 4 Centro de Investigación Biomédica en Red de Fisiopatología de la Obesidad y la Nutrición (CIBEROBN), Instituto de Salud Carlos III, 28029 Madrid, Spain

**Keywords:** fiber, immune homeostasis, microbiota, polyphenols, rats, short-chain fatty acids

## Abstract

**Background/Objectives**: Dietary fiber and polyphenols are recognized modulators of intestinal and immune homeostasis; however, evidence regarding their combined impact under physiological conditions remains limited. This study aimed to evaluate whether a diet enriched with fermentable fiber and polyphenols modulates mucosal and systemic immune biomarkers, as well as microbiota composition and function in healthy adult female rats. **Methods**: Wistar rats were fed either a reference diet (REF group) or a fiber- and polyphenol-enriched diet (FP group) for nine weeks. At the end of the intervention, plasma lipid profile, systemic and mucosal immune status (assessed by immunoglobulin (Ig) content in several compartments), cecal microbiota composition (determined by 16S rRNA sequencing), cecal short-chain fatty acids (SCFAs) and cecal Ig-coated bacteria, among other variables, were quantified. **Results**: The FP group exhibited a higher IgG concentration in plasma and elevated IgG2c levels in mucosal compartments compared with REF animals. The FP diet did not alter either intestinal morphology or hematologic and lipid variables; however, FP rats exhibited increased fecal moisture and reduced fecal pH. With regard to cecal microbiota, the FP group displayed higher microbial evenness, distinct β-diversity clustering, and shifts in the abundance of multiple genera. In addition, elevated cecal SCFA concentrations, particularly for acetate and propionate, were found in the FP group. **Conclusions**: Long-term intake of fermentable fiber and polyphenols promotes microbial fermentation and enhances humoral immunity without inducing structural or systemic physiological alterations. These findings support the role of plant-based foods in promoting immune and gut microbiota homeostasis under healthy conditions.

## 1. Introduction

It is well established that both gut microbiota and gut-associated lymphoid tissue (GALT) are modified by diet [1], and dietary fiber and polyphenols are bioactive compounds capable of modulating these interconnected systems.

Dietary fiber, composed of nondigestible carbohydrates (e.g., inulin and pectin), influences both gut ecology and immune function. In addition to its direct effects on the composition of microbiota, microbial fermentation of fiber produces short-chain fatty acids (SCFAs), which act as signaling molecules linking diet to host physiology [2]. SCFAs promote the differentiation of regulatory T (Treg) cells and enhance mucosal barrier integrity, among other functions [2,3]. Clinical studies have also shown that a high fiber intake is associated with lower levels of inflammatory markers, such as C-reactive protein [4]. Moreover, fiber intake modulates immune signaling pathways [3,4].

Polyphenols, mainly including flavonoids, and also phenolic acids and lignans, provide an additional nutritional strategy for modulating immunity. These compounds exert antioxidant and anti-inflammatory effects by influencing immune cell activation and cytokine production [5]. Polyphenols also affect gut microbiota by serving as substrates for beneficial microbes, counteracting deleterious microorganisms, and altering microbial composition toward health-promoting communities [6]. In turn, gut microbes biotransform polyphenols into bioactive metabolites that further modulate immune responses. Preclinical studies have shown that polyphenol-rich diets can skew dendritic cells toward a tolerogenic phenotype, increase the proportion of Treg cell populations, and suppress allergic-type inflammation [7,8]. In rodent models, hesperidin, a flavonoid found predominantly in citrus fruits [9], modulates both systemic and intestinal immune status [10], while cocoa polyphenol-enriched extracts modify immune cell populations and the composition of gut microbiota [11]. Collectively, these findings indicate that long-term dietary intake of fiber and polyphenols can prime the immune system even under physiological (nonchallenged) conditions.

In the context of maternal nutrition, the intake of fiber and polyphenols during gestation and lactation can modulate not only maternal but also the neonatal gut microbiota. Moreover, these dietary components influence the availability of microbial metabolites such as SCFAs and, consequently, shape early immune development in the offspring [12,13,14]. Therefore, ensuring an adequate maternal intake of fermentable fibers and polyphenols during critical developmental windows may represent an effective immunonutrition strategy in promoting immune homeostasis and reducing maternal alterations during gestation and lactation or even inflammatory predisposition in the next generation [12,14,15].

Despite growing evidence regarding the immunomodulatory effects of dietary fiber and polyphenols, the impact of their combination on immune and intestinal function in healthy adult females remains poorly characterized under controlled conditions. To date, most studies have focused on these bioactive compounds in the context of metabolic disorders, inflammatory conditions, or immunological challenges, leaving a gap in understanding how such diets influence immune homeostasis under physiological conditions. We hypothesized that, even in healthy individuals, a diet enriched with fermentable fiber and polyphenols beneficially modulates mucosal and systemic immune biomarkers, as well as the composition of microbiota in healthy adult females. Therefore, the aim of the present study was to evaluate the effects of a diet enriched with fermentable fibers (inulin and pectin) and a mixture of polyphenols (quercetin, naringenin, hesperidin, catechin, and epicatechin) on mucosal and systemic immunity, as well as the composition of cecal microbiota, in adult female Wistar rats.

## 2. Materials and Methods

### 2.1. Animals

Female Wistar rats (*n* = 8) were purchased from Janvier Labs (Saint-Berthevin, France; six weeks old on arrival). The animals were housed, in cages containing 2 animals each, in the experimental facility of the Diagonal Campus, Faculty of Pharmacy and Food Science, University of Barcelona (UB). Prior to the beginning of the study, the animals underwent a one-week acclimatization period. Housing conditions were standardized, with the temperature maintained at 21 ± 2 °C, a relative humidity of 50–55%, and a 12 h light/dark cycle. All experimental procedures were conducted in accordance with institutional ethical standards and approved by the UB Ethics Committee for Animal Experimentation (Ref. 162/25). The required sample size (*n* = 4 per group) was calculated using the G*Power software, version 3.1.9.7 (Heinrich-Heine-Universität Düsseldorf, Düsseldorf, Germany). The calculation was based on intestinal IgA, among other variables, assuming no dropout rate and a two-sided type I error of 0.05. Although the 3Rs sustained this approach and given the exploratory nature of the study and the expected variability of microbiota and immune-related parameters, the statistical power may be insufficient for detecting moderate effects.

### 2.2. Diets and Study Design

Two diets were supplied by Envigo^®^—Teklad Diets (Madison, WI, USA). A fiber- and polyphenol-enriched diet (FP) consisted of 8% chicory inulin, 1% pectin, and 0.5% of a polyphenolic blend (catechin, epicatechin, hesperidin, naringenin, and quercetin; Sigma-Aldrich^®^, Madrid, Spain), while a standard AIN-93G formulation was used as the control diet [16]. Both diets had the same protein and fat content by weight, while the FP diet had a slightly lower energy density than the reference diet, 3.5 vs. 3.7 kcal/g. Animals (*n* = 4 per group) were randomly assigned to either the FP or REF group and were fed the corresponding diet for nine weeks, with food and water available ad libitum.

### 2.3. Monitoring, Sample Collection and Processing

Body weight, water intake, and food consumption were monitored weekly throughout the experimental period. In addition, fecal samples were collected weekly to determine pH and moisture content. A portion of fresh fecal material was used for determining pH using a surface electrode (Crison Instruments, S.A., Barcelona, Spain), and fecal moisture content was calculated by weighing the samples before and after drying at 60 °C for 24 h.

After nine weeks of the dietary intervention, the animals were anesthetized with ketamine (90 mg/kg) and xylazine (10 mg/kg) (Bayer A.G., Leverkusen, Germany). Measurements required to calculate body mass index (BMI, weight/length^2^ (g/cm^2^)) and the Lee index ((weight^0.33^/length) × 1000 (g^0.33^/cm)) were obtained.

Blood samples were collected via cardiac exsanguination in EDTA-containing tubes and analyzed using an automated hematology analyzer (Spincell; MonLab Laboratories, Barcelona, Spain). Plasma was subsequently separated and stored at −20 °C and −80 °C until analysis. The mesenteric lymph nodes (MLNs), thymus, spleen, liver, brain, heart, empty stomach, kidney, submaxillary salivary glands, small intestine (SI), cecum, and colon were excised. Tissues were weighed and, depending on the subsequent analyses, either processed immediately or stored at −20 °C or −80 °C. A fraction of the MLNs was kept at −20 °C and subsequently homogenized following established protocols [17].

The SI was processed for several purposes. To obtain gut wash (GW), a proximal segment of about 10 cm was opened longitudinally, cut into 0.5 cm pieces, weighed, and incubated in PBS at 37 °C under agitation for 10 min, following previously described procedures [18]. In addition, a 1 cm fragment from the middle portion of the SI was collected for histomorphometric studies. An adjacent 1 cm fragment was collected for gene expression analysis; this was immersed in RNAlater (Ambion, Life Technologies, Madrid, Spain), maintained at 4 °C for 24 h, and subsequently stored at −20 °C. Moreover, cecal content (CC) was collected for microbiota analysis.

### 2.4. Lipidomic Study

Concentrations of triglycerides (TG), total cholesterol (TC), and high-density lipoprotein cholesterol (HDL-C) were quantified in plasma samples stored at −80 °C and thawed at 4 °C, with commercially available assay kits from Química Clínica Aplicada (QCA, Amposta, Spain) being used for this purpose. TG, TC, and HDL-C concentrations were quantified using the glycerol phosphate dehydrogenase (GPO) method, the cholesterol oxidase-peroxidase (CHOD-POD) method, and a colorimetric assay, respectively. Low-density lipoprotein cholesterol (LDL-C) concentration was estimated using the Friedewald formula [19].

### 2.5. Quantification of Ig Concentrations

The concentrations of IgA, IgM, IgG1, IgG2a, IgG2b, and IgG2c were measured in plasma, GW, and homogenized MLNs using a ProcartaPlex™ Multiplex immunoassay kit (Thermo Fisher Scientific, Vienna, Austria), following previously established protocols [20]. Ig quantification was performed using a MAGPIX^®^ analyzer (Luminex Corporation, Austin, TX, USA) at the Scientific and Technological Centers of the University of Barcelona (CCiT-UB). Assay sensitivities were 0.15 ng/mL for IgM, 1.16 ng/mL for IgG1, 2.08 ng/mL for IgG2a, 2.68 ng/mL for IgG2b, 4.21 ng/mL for IgG2c, and 0.46 ng/mL for IgA. Ig concentrations in MLN homogenates were expressed as ng/g of tissue and in GW samples as ng/mL of diluted sample.

The secretory IgA (sIgA) concentration in MLN homogenates and GW was determined by sandwich ELISA (Bethyl Laboratories Inc., Montgomery, AL, USA), as previously described [21].

### 2.6. Intestinal Histology

SI sections were fixed in 4% buffered formaldehyde (24 h), washed in PBS (3 h), dehydrated through a graded ethanol series (70%, 90%, and 100%), and subsequently cleared in xylene (Panreac Química SLU, Barcelona, Spain), with samples then being embedded in paraffin (Merck, Madrid, Spain). Paraffin blocks were sectioned at a thickness of 5 µm, and the sections were stained with hematoxylin-eosin as previously described [22].

Histological evaluation was carried out using a light microscope (Olympus BX41) equipped with a digital camera (Olympus XC50, Olympus, Barcelona, Spain). Representative micrographs were obtained at 10× magnification and analyzed with ImageJ software, version 1.54f (National Institute of Mental Health, Bethesda, MD, USA). For each animal, ten villi were randomly selected for morphological assessment, and measurements included villus height, width, and area. Villus width was measured at the crypt–villus junction, whereas villus area was calculated by delineating the region of interest excluding the crypts. Crypt depth and the number of goblet cells per villus were also determined, and the villus height-to-crypt depth was calculated.

### 2.7. Gene Expression Analysis

SI samples stored in RNAlater were subjected to RNA extraction. Briefly, samples were placed into lysing matrix tubes (MP Biomedicals, Illkirch, France) and homogenized using a FastPrep-24 instrument (MP Biomedicals). An RNeasy Mini Kit (Qiagen, Madrid, Spain) was used for extracting RNA according to the manufacturer’s instructions. RNA purity and concentration were determined using a NanoPhotometer (BioNova Scientific S.L., Fremont, CA, USA), and cDNA was obtained using TaqMan Reverse Transcription Reagents (Applied Biosystems, AB, Weiterstadt, Germany).

Target genes used for PCR are listed in Supplementary Table S1. Real-time (RT)-PCR was performed utilizing the ABI Prism 7900 HT quantitative RT-PCR system (AB). Gene expression levels were normalized to the housekeeping gene *Gusb* (β-glucuronidase) and analyzed using the 2^−ΔΔCt^ method, as previously described [23]. Data are expressed as the percentage of gene expression in each experimental group relative to the mean value obtained of the REF group, which was set to 100%.

### 2.8. Cecal Bacteria and Ig-Coated Bacteria Analyses

The proportion of cecal bacteria and Ig-coated bacteria (Ig-CB) in CC was determined by flow cytometry, as previously described [24]. Briefly, the cecal homogenates were prepared following previously described procedures [25] and subsequently stained with a fluorescein isothiocyanate (FITC)-conjugated rabbit anti-rat Ig antibody (Abcam, Cambridge, UK) and with propidium iodide (PI) (Merck KGaA). Bacteria were gated using an Aurora Cytek spectral flow cytometer (Cytek, Fremont, CA, USA) based on forward scatter (FSC), side scatter (SSC), and PI staining characteristics. Data were acquired in the Flow Cytometry Unit of the CCiT-UB, and data analysis was conducted using FlowJo v.10 software (Tree Star, Inc., Ashland, OR, USA).

### 2.9. Microbiota Analysis

From CC samples, the V3–V4 region of the 16S rRNA gene was amplified using 25 PCR cycles. Negative extraction controls and a positive Mock Community control were included to ensure reliability of sequencing. Libraries were sequenced using the Illumina MiSeq platform with a 2 × 300 bp paired-end configuration (Illumina Inc., San Diego, CA, USA). Sequence merging and processing were performed using the MiSeq Run and MiSeq Reporter software (version 4.1.0) developed by Microomics Systems S.L. (Barcelona, Spain).

Microbial diversity analyses were conducted to characterize within- and between-group community differences. Alpha diversity was evaluated based on the number of observed operational taxonomic units (OTUs, representing richness) and Pielou’s Evenness Index. Beta diversity was assessed using the Jaccard distance to account for compositional dissimilarity between groups. Taxonomic assignment of phylotypes was achieved through a Bayesian classifier trained with the SILVA database (version 132; 99% OTUs, full-length sequences) [26]. Relative abundances of bacterial families and genera were visualized as stacked bar plots. To identify bacterial taxa differentially associated with each experimental group, a linear discriminant analysis (LDA) was performed based on relative abundance data at the genus level. This analysis enabled the detection of microbial features that most strongly explained the differences between groups by estimating their effect size.

### 2.10. Short-Chain Fatty Acid Quantification

SCFAs were quantified by gas chromatography–mass spectrometry (GC–MS) as previously described [27], with slight modifications. Samples were supplemented with an internal standard (3-methylvaleric acid), processed, and centrifuged (1500× *g*, 2 min, 4 °C). The resulting supernatant was collected, filtered through a 0.22 μm PES membrane (Sarstedt SA, Nümbrecht, Germany), and subsequently analyzed using an Agilent 7890B–5977B GC–MS system equipped with a multipurpose sampler (Gerstel MPS, Mülheim, Germany). Separation was achieved on an Agilent DB-FATWAX column (30 m × 0.25 mm × 0.25 μm) operated in split mode (20:1). The oven temperature program was set as follows: initial temperature of 100 °C for 3 min, increased to 150 °C at 5 °C min^−1^ and held for 1 min, then raised to 200 °C at 20 °C min^−1^, and maintained for 5 min. Helium served as the carrier gas at a flow rate of 1 mL min^−1^, with the injector temperature maintained at 250 °C. A 2 μL aliquot was injected for each sample. Calibration curves prepared with acetate, propionate, and butyrate standards were used for quantifying SCFA.

### 2.11. Statistical Analysis

Statistical analyses were performed using SPSS Statistics version 22.0 (SPSS Inc., Chicago, IL, USA). Normally distributed data were analyzed using Student’s *t*-test, and nonnormally distributed with the Mann–Whitney U test. For variables involving multiple comparisons, i.e., fecal moisture, fecal pH, and body weight, a Bonferroni correction was applied to adjust the significance level. Differences were considered statistically significant at *p* < 0.05.

To evaluate differences in microbial composition between the FP and REF groups, α-diversity indices (observed OTUs and Pielou’s evenness) were compared using the nonparametric Mann–Whitney U test. Beta diversity was explored through principal coordinates analysis (PCoA) based on Jaccard distance matrices generated using R software (version 3.6.0). Group clustering significance was assessed using PERMANOVA and ANOSIM analyses, while PERMDISP was applied to distinguish whether observed differences were attributable to group location or dispersion [28]. Differences in the relative abundance of bacterial taxa were evaluated using two complementary approaches: ANCOM, to account for compositional data characteristics [29], and Mann–Whitney U tests for direct pairwise comparisons.

Correlations between relative bacterial abundances, Ig concentrations and SCFA levels were assessed using Spearman’s rank correlation analysis in SPSS software version 22.0. Correlation coefficients of (ρ) ≥ 0.7 or ≤ −0.7 were considered indicative of strong associations.

No inclusion or exclusion criteria were predefined, and no animals or data points were excluded from the analyses. Blinding was not performed during the experiments and data analysis.

## 3. Results

### 3.1. Morphometric Variables

**Figure 1:**
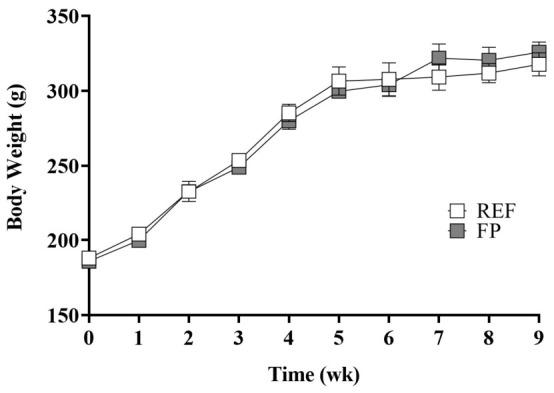
Body weight over 9 weeks of diet. REF group: animals fed the standard diet; FP group: animals fed the fiber- and polyphenol-enriched diet. Results are expressed as mean ± SEM (*n* = 4).

Body weight (BW) was monitored weekly throughout the 9-week dietary intervention, during which both groups exhibited a normal growth time course consistent with age. No significant differences in BW were observed between rats in the FP group and those in the REF group at any point during the study (Figure 1).

At the end of the 9-week intervention, morphometric parameters were assessed and no significant differences were found between the FP and REF groups. Similarly, most relative organ weights were comparable between the two groups. However, rats fed the FP diet displayed significantly higher relative stomach and cecum tissue weights than those in the REF group (Table 1).

### 3.2. Hematologic and Lipidomic Variables

Results regarding hematological variables and plasma lipid profile (Table 2) show no significant differences between the FP and REF groups.

### 3.3. Immunoglobulin Concentration

**Figure 2:**
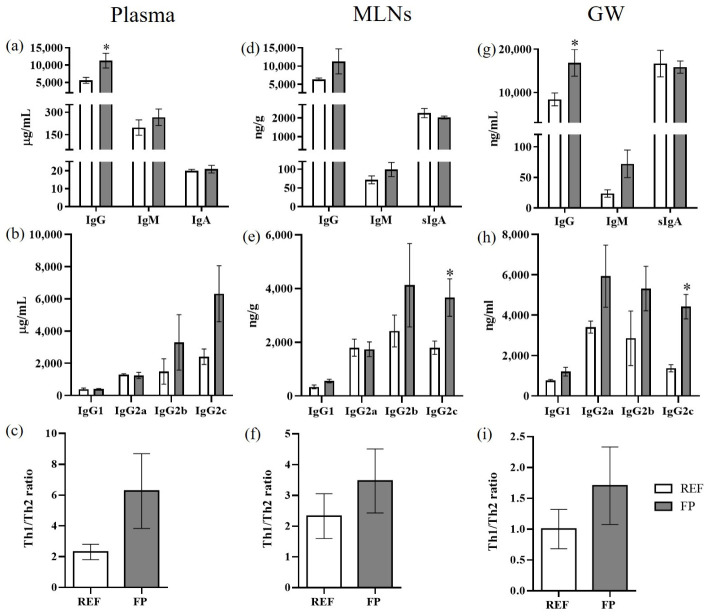
Ig concentrations in plasma, mesenteric lymph node (MLN) homogenates, and gut wash (GW) samples: main Igs (**a**,**d**,**g**), IgG isotypes (**b**,**e**,**h**) and ratio between Th1- and Th2-associated IgG isotypes (**c**,**f**,**i**). REF group was fed the standard diet; FP group was fed the fiber- and polyphenol-enriched diet. Data are expressed as mean ± SEM (*n* = 4). Statistical differences: * *p* < 0.05 vs. reference group.

To assess systemic and mucosal humoral immunity, Ig concentrations were analyzed in plasma, MLN homogenates, and GW samples (Figure 2).

In plasma (Figure 2a–c), animals from the FP group exhibited significantly higher total IgG concentrations than those in the REF group, which can be attributed to higher IgG2b and IgG2c concentrations induced by the FP diet. No changes were observed for any other Ig. Given that, in rats, IgG2b and IgG2c are associated with a Th1-type immune response, whereas IgG1 and IgG2a are linked to a Th2-type response [30], no significant difference was observed either in the Th1/Th2 ratio.

In MLNs (Figure 2d–f), IgG, IgM and IgA concentrations were comparable between the two groups. However, IgG2c content was significantly higher in the FP group than in the REF group (Figure 2e). The Th1/Th2-associated IgG subclass ratio also remained unchanged between diets.

Finally, in the GW samples (Figure 2g–i), rats from the FP group exhibited a higher IgG concentration than those in the REF group (Figure 2g), which can be attributed to IgG2a, IgG2b, and, above all, to IgG2c content (Figure 2h). No differences were detected for any other Ig (Figure 2g) or for the Th1/Th2-associated IgG subclass ratio.

### 3.4. Fecal Moisture and pH

**Figure 3:**
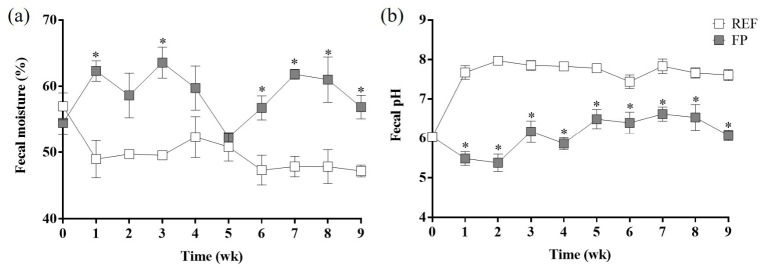
Fecal moisture (**a**) and pH (**b**) throughout the 9 weeks of the diet. REF group was fed the standard diet; FP group was fed the fiber- and polyphenol-enriched diet. Data are expressed as mean ± SEM (*n* = 4). Statistical differences: * *p* < 0.05 vs. reference group.

Fecal moisture and pH were monitored weekly throughout the 9-week dietary intervention. Overall, the FP group displayed higher fecal moisture than the REF group, with mean values of approximately 60% and 50%, respectively (Figure 3a). This change was observed after just one week of the diet. In addition, fecal pH was significantly lower in the FP group than in the REF group from the first week of the study. Values in the FP group remained stable at around pH 6, whereas in the REF group values were close to pH 7.8 throughout the experimental period (Figure 3b).

### 3.5. Small Intestine Morphology and Gene Expression

**Figure 4:**
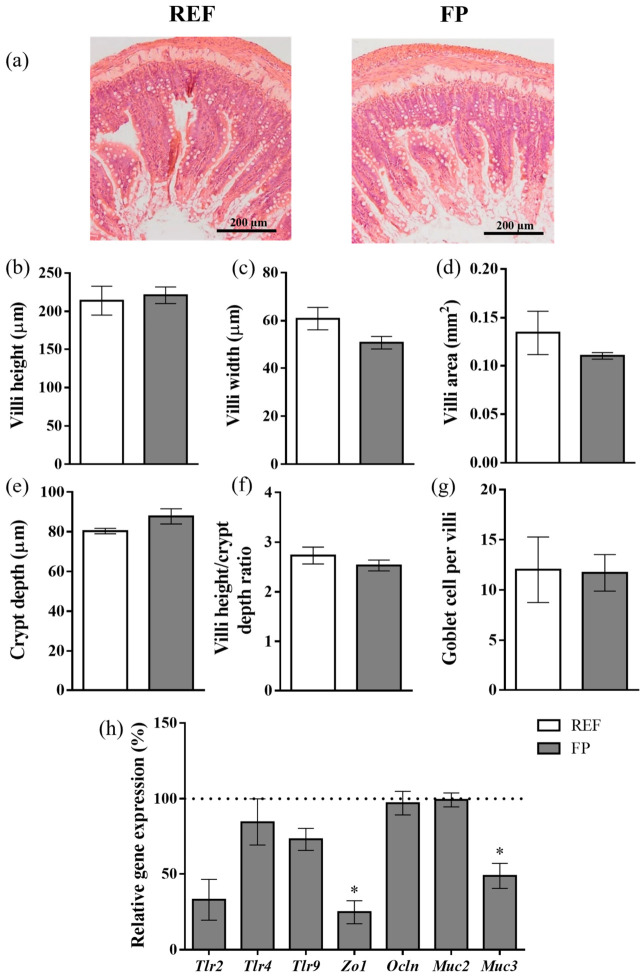
Small intestine variables after 9 weeks of diet. (**a**) Representative SI sections stained with hematoxylin and eosin, 10×; (**b**) villi height; (**c**) villi width; (**d**) villi area; (**e**) crypt depth; (**f**) villi/crypt ratio; (**g**) number of goblet cells per villus; (**h**) modulation of gene expression in the SI (percentage of expression normalized to the mean value obtained for the REF group, set as 100%, indicated by a dotted line). REF group was fed the standard diet; FP group was fed the fiber- and polyphenol-enriched diet. Data are expressed as mean ± SEM (*n* = 4). Statistical differences: * *p* < 0.05 vs. reference group.

SI morphology was evaluated through histological analysis to assess the effects of the FP diet on intestinal architecture (Figure 4a–g). No differences were observed between the two groups in any of the variables considered including the content of goblet cells.

In addition, the relative expression of *Tlr2*, *Tlr4*, *Tlr9*, *ZO-1*, *Ocln*, *Muc2*, and *Muc3* genes was assessed (Figure 4h), with a significant reduction in *ZO-1* and *Muc3* gene expression being observed in rats from the FP group compared with those in the REF group.

### 3.6. Number of Total Bacteria and Ig-Coated Bacteria in the Cecum

**Figure 5:**
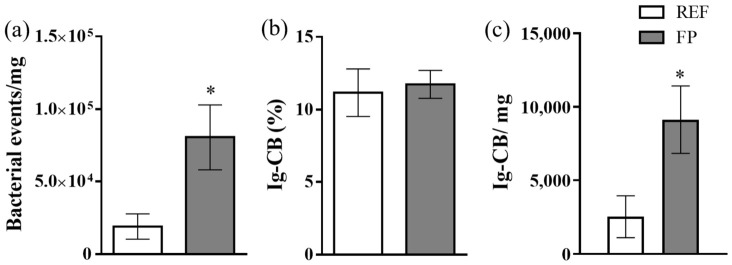
Number of bacteria and relative and absolute number of Ig-CB after 9 weeks of diet. (**a**) Total bacteria counts in the CC; (**b**) proportion of Ig-CB in the CC; (**c**) total counts of Ig-CB in the CC. REF group was fed the standard diet; FP group was fed the fiber- and polyphenol-enriched diet. Data are expressed as mean ± SEM (*n* = 4). Statistical differences: * *p* < 0.05 vs. reference group.

After 9 weeks, the FP diet was associated with an increase in the number of total cecal bacteria (Figure 5a), whereas the proportion of cecal Ig-CB remained stable between the two groups (Figure 5b). Consequently, there was a higher number of Ig-CB in the FP group than in the REF group (Figure 5c).

### 3.7. Microbiota Composition

**Figure 6:**
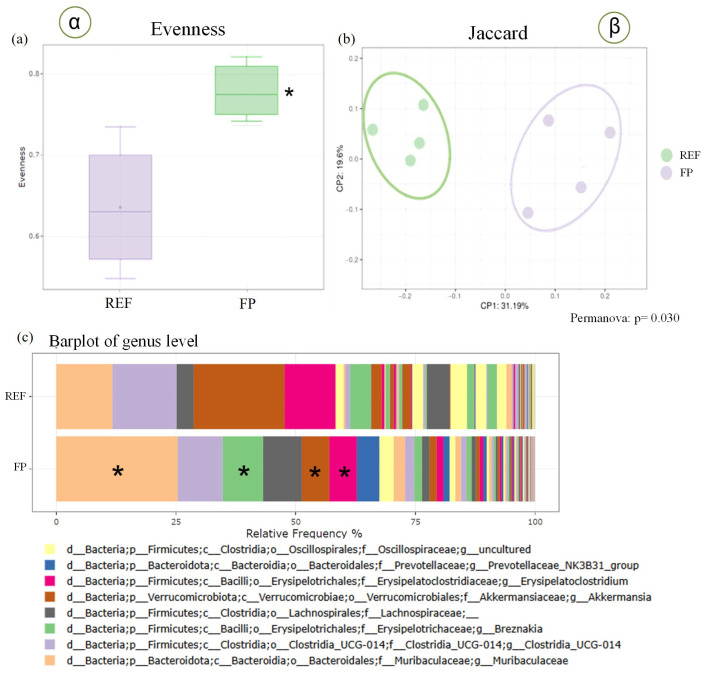
Cecal microbiota diversity and composition after 9 weeks of diet. Alpha-diversity index (Evenness) (**a**) and β-diversity index (Jaccard) (**b**). Relative genus abundance (**c**). Statistical testing was performed by PERMANOVA using Bray–Curtis distances, and the Mann–Whitney test was used for α-diversity index. REF group was fed the standard diet; FP group was fed the fiber- and polyphenol-enriched diet. Results are expressed as mean relative abundance (*n* = 4). Statistical differences: * *p* < 0.05 vs. reference group.

The composition of cecal microbiota after 9 weeks of the diet revealed significant differences between the two groups (Figure 6). In terms of α-diversity, there was a higher evenness index, which is indicative of microbial diversity, in the FP group than in the REF group. Moreover, β-diversity analysis, estimated using the Jaccard Index indicated clear differences in the microbial community structure between the two groups. The PERMANOVA test confirmed that the FP diet had a significant influence on microbial composition (*p* = 0.03).

With respect to taxonomic analysis, differences in the proportions of bacterial genera were identified between the groups. Among the most abundant genera, the FP diet led to higher proportions of *Muribaculaceae* and *Breznakia* than the REF diet, whereas lower proportions of *Akkermansia* and *Erysipelatoclostridium* were observed. In addition, in less represented genera, an increase in the proportion of *Ruminococcus* and a decrease in *Lactococcus* were found in the FP group.

**Figure 7:**
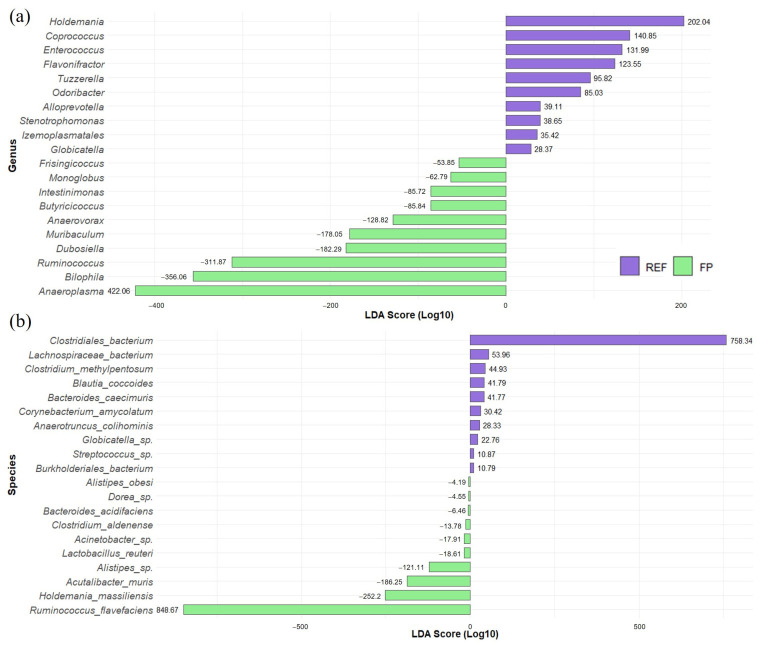
Linear discriminant analysis (LDA) effect size of the CC microbiota size identifying discriminant genus (**a**) and species (**b**) between the REF and FP diets, after 9 weeks of diet. LDA results are expressed as log^10^-transformed LDA scores. REF group was fed the standard diet; FP group was fed the fiber- and polyphenol-enriched diet.

LDA revealed clear diet-associated microbial signatures at both the genus and species levels (Figure 7). As regards genus (Figure 7a), the REF group was characterized by higher abundances of *Holdemania*, *Coprococcus*, and *Enterococcus*, whereas the FP group was discriminated by *Anaeroplasma*, *Bilophila*, and *Ruminococcus.* As for species (Figure 7b), the REF group was primarily driven by *Clostridiales bacterium*, while the FP group was most strongly discriminated by *Ruminococcus flavefaciens*, which displayed the highest LDA score overall in this group. The enrichment of *Ruminococcus* at both genus and species levels highlights this taxon as a key microbial signature induced by the FP diet.

### 3.8. Cecal Short-Chain Fatty Acids Profile

**Figure 8:**
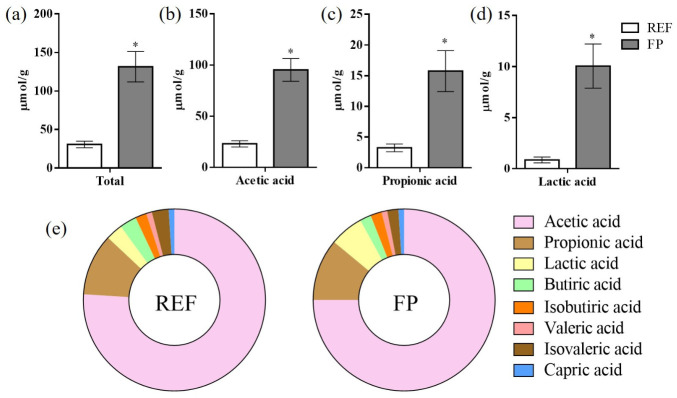
Short-chain fatty acids analysis in cecal content after 9 weeks of diet. Concentrations of (**a**) total, (**b**) acetic, (**c**) propionic, and (**d**) lactic acids, and (**e**) relative proportion of each SCFA. REF group was fed the standard diet; FP group was fed the fiber- and polyphenol-enriched diet. Results are expressed as mean ± SEM (*n* = 4). Statistical differences: * *p* < 0.05 vs. reference diet.

The analysis of SCFA concentrations in the cecum after 9 weeks of FP diet revealed that FP rats showed markedly higher concentrations of SCFA, with higher contents of acetic, propionic, and lactic acids than the REF group (Figure 8a–d). This increase similarly affected the other SCFAs because, when comparing their proportions (Figure 8e), no significant differences were observed between the FP and REF groups.

### 3.9. Correlations Between Studied Variables

**Figure 9:**
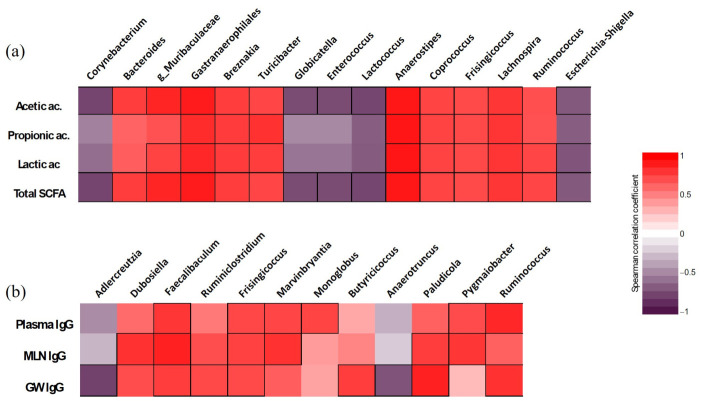
Heatmap of Spearman correlations between selected bacterial taxa and SCFA (**a**) and IgG concentrations in several compartments (**b**). The Spearman correlation coefficient is represented on the heatmap following the color in the legend. Boxed squares represent correlations with statistical significance (*p* < 0.05).

Correlation analysis revealed that the concentrations of acetic acid, as well as total SCFAs, were positively associated with several FP-enriched taxa, including the genus *Muribaculaceae* and *Ruminococcus*, while negative correlations were observed with taxa such as *Lactococcus*, found in lower proportions in the FP group and also with the proportions of *Enterococcus* and *Escherichia/Shigella* (Figure 9a).

On the other hand, IgG concentrations in plasma, MLNs, and GW showed significantly positive associations with some genera, including *Ruminococcus* (Figure 9b).

## 4. Discussion

The present study demonstrates that long-term consumption of a fiber- and polyphenol-enriched (FP) diet under physiological conditions is well tolerated in adult rats and confers immunological and intestinal benefits without compromising systemic health.

The absence of alterations in body weight gain, hematological variables, lipid profile, and intestinal morphology confirms the safety of this dietary pattern in healthy adult female rats. These findings are consistent with previous studies reporting that diets enriched with fermentable fibers such as inulin or pectin do not induce adverse metabolic or toxicological effects when administered chronically [31,32]. Likewise, polyphenols administered in nutritionally relevant doses are generally considered safe, with both preclinical and clinical evidence indicating good tolerability and an absence of deleterious systemic effects [5,6].

In addition to its safety profile, the present study revealed an upregulation of humoral immune responses, particularly an increase in plasma IgG concentrations, mainly due to IgG2b and IgG2c levels, as well as enhanced intestinal IgG evidenced by higher IgG2c concentrations in GW and MLNs. These findings were observed in healthy rats in the absence of an immune challenge, highlighting the capacity of the dietary fibers and polyphenols used in this study to enhance humoral defense at both mucosal and systemic levels. These results are consistent with the increase in serum IgG concentration reported in piglets supplemented with licorice flavonoids powder for 5 weeks [33]. In addition, supplementation with 400 mg/kg dandelion flavonoids significantly increased serum IgG, IgM and IgA levels in lipopolysaccharide (LPS)-stressed broilers [34]. Similarly, quercetin supplementation enhanced the IgG concentration in growing pigs challenged with *Escherichia coli* LPS [35]. It has also been reported that dietary fiber has modulated circulating and intestinal IgG concentrations in animal models. In growing pigs, a high-fiber diet increased ileal IgG concentrations in the Duroc strain [36]. Maternal supplementation with a fiber mixture of β-glucan and fructo-oligosaccharides increased serum IgG concentrations in both sows and piglets, while also raising IgG levels in colostrum [37]. Similarly, wheat bran supplementation increased serum IgG concentrations in weaned piglets compared with sugar beet pulp at day 14 [38]. Our data extend these observations by demonstrating that the combined administration of fermentable fibers and mixed flavonoids promotes IgG production in both systemic and mucosal compartments in healthy adult females. These findings are particularly relevant in the context of gestating mothers. Maternal IgG represents the main Ig class transferred to offspring, primarily through the placenta but also via breast milk, thus contributing to neonatal immune protection during early life [39,40]. Recent evidence further indicates that maternal IgG can shape the composition of neonatal gut microbiota by coating commensal bacteria transmitted through milk, thereby influencing immune education in the offspring [41]. Thus, a diet capable of enhancing maternal IgG levels could potentially improve not only passive immune transfer but also early-life immune programming.

In regard to epithelial barrier-related markers, the FP diet selectively reduced *ZO-1* and *Muc3* gene expression, while *Ocln*, *Muc2* and the assessed *Tlr* genes remained unchanged. It has been stated that *ZO-1* is essential for mucosal repair, whereas soluble fiber intake in rats reduced ileal *Muc3* gene expression despite increasing luminal mucin content [42,43]. However, the reductions in *ZO-1* and *Muc3* gene expression should be interpreted cautiously because transcriptional changes were not accompanied with intestinal permeability and mucus layer integrity assessment.

In parallel with the enhancement of humoral immunity, the FP diet increased the total number of cecal bacteria and modified both microbiota composition and activity. Increased α-diversity evenness and distinct β-diversity clustering indicate that the FP diet reshaped the microbial ecosystem. Notably, greater evenness is considered a marker of gut ecosystem resilience and functional stability [44,45]. In parallel, the FP diet promoted increased cecal SCFA production that reduced fecal pH. Although fecal pH decreased from the first week of intervention and SCFA concentrations were only determined at week 9 in cecal content, the reduced fecal pH at the end of the study is consistent with the higher SCFA production observed in the cecal content. These changes were accompanied by increased cecal weight in FP-fed rats, which may reflect an adaptive response to enhanced luminal bulk and microbial fermentation, as previously reported in pigs [46]. Similar findings have been described in rodents receiving fermentable substrates, in which cecal hypertrophy is associated with enhanced microbial fermentation and increased SCFA production [47,48,49].

Analysis at the bacterial genus level revealed enrichment of fiber-degrading and SCFA-producing genera, including *Muribaculaceae* and *Ruminococcus*, together with a reduced abundance of mucus-degrading taxa such as *Akkermansia*. These findings suggest there is a shift toward a microbiota optimized for fermentative metabolism [50,51]. Similarly, LDA identified *Ruminococcus* as a key discriminant genus enriched in the FP group. Correlation analyses showed that lactic acid and total SCFA concentrations were positively associated with *Ruminococcus*, thus confirming its contribution to enhanced fermentative activity. Members of the *Ruminococcus* genus are recognized for their ability to degrade resistant starch and other complex carbohydrates in the colon, thereby initiating fiber breakdown and releasing fermentable substrates that support broader SCFA-producing networks [52,53]. These compositional changes are consistent with previous reports showing that polyphenols selectively promote saccharolytic bacteria while limiting potentially detrimental taxa [54,55,56].

The higher relative abundance of *Ruminococcus* in the FP group correlated with both systemic and mucosal IgG levels, suggesting that its fermentative activity and the resulting SCFA production may contribute to enhancing immunity [57]. Collectively, these results indicate that the FP diet not only reshapes the microbial community structure but also promotes functionally relevant taxa, with *Ruminococcus* acting as a central node linking dietary fibers, SCFA production, and host immune responses.

The increase in SCFA and lactic acid production caused by the FP diet negatively correlated with genera such as *Enterococcus* and *Escherichia/Shigella*, which have been associated with dysbiosis and pro-inflammatory states [58,59]. Dysbiosis and reduced microbial diversity have been linked to disorders affecting female reproductive health and fertility, including polycystic ovary syndrome, endometriosis, and unexplained infertility in both humans and animal models [60,61]. The restoration of microbial balance or supplementation with SCFAs has been shown to improve ovarian function, follicular development, and reproductive outcomes in rodents and livestock [62,63]. Although reproductive endpoints were not evaluated in the present study, the FP-induced enhancement of microbial diversity and SCFA production suggests a healthy status that favors reproductive fitness.

From a broader perspective, these findings support the concept that pregestational period represents a valuable window during which dietary interventions may optimize maternal immune and microbial status before gestation and lactation.

The present study has several limitations. First, blinding was not performed during the experiment and data analysis, which could be considered a methodological limitation. Moreover, the relatively small sample size restricts the statistical power and generalizability of the findings and may be insufficient to detect moderate effects, particularly for high-variability outcomes such as microbiota composition, immune biomarkers, gene expression, and correlation analyses. Therefore, some nonsignificant findings may reflect type II errors, and correlation analyses should be interpreted cautiously as exploratory and hypothesis generating due to the limited number of observations. In addition, although the 9-week intervention period does not allow conclusions to be drawn regarding long-term effects, it is consistent with previous nutritional studies in rats reporting diet-induced changes in gut microbiota composition, cecal fermentation, fecal characteristics, and immune-related parameters after even shorter dietary exposures [64,65,66,67]. Thus, the present findings should be interpreted as preliminary evidence of the effects of the FP diet during the analyzed period. Although both diets had the same protein and fat content, the FP diet had a slightly lower energy density than the reference one due to the inclusion of fermentable fibers and polyphenols; therefore, potential effects derived from differences in energy density cannot be completely excluded. In addition, because the FP intervention combined fermentable fibers and polyphenols within a single dietary treatment, it is not possible to determine whether the observed effects were mainly driven by the fiber fraction, the polyphenol fraction, or by additive or synergistic interactions between the two components. Finally, the study was only conducted in female rats, and therefore the applicability of the findings to males remains uncertain, as sex-dependent differences in immune responses, gut microbiota composition, intestinal fermentation, and SCFA production may influence the physiological response to dietary fiber and polyphenol interventions.

## 5. Conclusions

In conclusion, a diet enriched with fermentable fibers and flavonoid polyphenols was safe and well tolerated among healthy adult female Wistar rats. This dietary intervention enhanced humoral immunity at both systemic and intestinal levels. In parallel, the diet promoted a more favorable intestinal environment, characterized by increased microbial diversity and enhanced SCFA production, reflecting improved microbial functionality during the analyzed period.

## Figures and Tables

**Table 1:** Morphometric variables after 9 weeks of diet.

Body	REF	FP
Final body weight (g)	317.75 ± 8.53	325.83 ± 18.52
BMI (g/cm^2^)	0.70 ± 0.03	0.66 ± 0.01
Lee index (g^0.33^/cm × 1000)	319.45 ± 5.61	310.57 ± 3.56
**Tissue/Body Weight (%)**		
Thymus	0.21 ± 0.02	0.17 ± 0.01
Spleen	0.25 ± 0.01	0.23 ± 0.00
Submaxillary salivary gland	0.17 ± 0.01	0.15 ± 0.01
Stomach	0.43 ± 0.01	0.52 ± 0.03 *
Small Intestine	2.25 ± 0.07	2.25 ± 0.19
Cecum	0.18 ± 0.03	0.35 ± 0.03 *
Liver	3.00 ± 0.15	3.36 ± 0.07
Kidney	0.33 ± 0.02	0.36 ± 0.01
Brain	0.52 ± 0.02	0.53 ± 0.02
Heart	0.33 ± 0.01	0.31 ± 0.01

REF group was fed the standard diet; FP group was fiber- and polyphenol-enriched diet. Data expressed as mean ± SEM (*n* = 4). Statistical differences: * *p* < 0.05 vs. reference group.

**Table 2:** Hematologic and lipidomic variables after 9 weeks of diet.

Hematologic		REF	FP
Leukocytes (×10^9^/L)		6.70 ± 0.50	6.00 ± 0.86
	• Lymphocytes (×10^9^/L)	2.20 ± 0.16	2.27 ± 0.24
	• Monocytes (×10^9^/L)	0.25 ± 0.03	0.25 ± 0.03
	• Granulocytes (×10^9^/L)	4.28 ± 0.51	3.50 ± 0.74
Erythrocytes (×10^12^/L)		7.61 ± 0.22	7.72 ± 0.41
Hemoglobin (HGB, g/L)		13.23 ± 0.19	13.20 ± 0.29
Hematocrit (HCT, %)		41.25 ± 1.59	43.33 ± 1.91
Mean corpuscular volume (MCV, fL)		54.28 ± 0.78	56.37 ± 1.16
Mean corpuscular HGB (MCH, pg)		17.45 ± 0.51	17.17 ± 0.61
Platelets (×10^9^/L)		463.25 ± 35.68	473.33 ± 69.91
**Lipidomic**			
Triglycerides (mg/dL)		76.73 ± 10.26	90.09 ± 11.11
Cholesterol (mg/dL)		87.17 ± 8.01	107.78 ± 10.36
High-density lipoprotein cholesterol (HDL-C, mg/dL)		27.55 ± 2.44	36.14 ± 5.38
Low-density lipoprotein cholesterol (LDL-C, mg/dL)		41.92 ± 6.43	56.32 ± 9.04

REF group was fed the standard diet; FP group was fiber- and polyphenol-enriched diet. Data are expressed as mean ± SEM (*n* = 4).

## Data Availability

The datasets generated and/or analyzed during the current study are available from the corresponding author on reasonable request. The data are not publicly available due to ongoing further research.

## Supplementary Materials

The following supporting information can be downloaded at https://www.mdpi.com/article/10.3390/nu18132088/s1. Table S1: Description of the specific TaqMan primers AB.
