# Supplementary material for: A Fiber- and Polyphenol-Enriched Diet Enhances Humoral Immunity, Reshapes Cecal Microbiota, and Improves Short-Chain Fatty Acid Production in Female Wistar Rats
Source: Nutrients. 2026 Jun 26;18(13):2088. doi: 10.3390/nu18132088 (PMC13363427; doi:10.3390/nu18132088)
Supplement: Supplementary file 1 [file nutrients-18-02088-s001.zip › nutrients-4374932-supplementary.pdf]

Supplementary Table S1. Description of the specific TaqMan primers AB

Gene	Reference
Toll-like receptor 2 *(Tlr2)*	Rn02133647_s1, I
Toll-like receptor 4 (*Tlr4)*	Rn00569848_m1, I
Toll-like receptor 9 *(Tlr9)*	Rn01640054_m1, I
Zonula occludens 1 (*ZO-1)*	Rn02116071_s1, I
Occludin *(Ocln)*	Rn00580064_m1, I
Mucin 2 (*Muc2)*	Rn01498206_m1, I
Mucin 3 (Muc3)	Rn01481134_m1, I
Glucuronidase beta (*Gusb)*	Rn00566655_m1, I

I, inventoried
